# Fat-to-glucose interconversion by hydrodynamic transfer of two glyoxylate cycle enzyme genes

**DOI:** 10.1186/1476-511X-7-49

**Published:** 2008-12-10

**Authors:** P Cordero, J Campion, FI Milagro, F Marzo, JA Martinez

**Affiliations:** 1 Department of Nutrition and Food Sciences, Physiology and Toxicology, University of Navarra, Pamplona, Spain; 2 Laboratory of Animal Physiology and Nutrition, School of Agronomy, Public University of Navarra, Pamplona, Spain

## Abstract

The glyoxylate cycle, which is well characterized in higher plants and some microorganisms but not in vertebrates, is able to bypass the citric acid cycle to achieve fat-to-carbohydrate interconversion. In this context, the hydrodynamic transfer of two glyoxylate cycle enzymes, such as isocytrate lyase (ICL) and malate synthase (MS), could accomplish the shift of using fat for the synthesis of glucose. Therefore, 20 mice weighing 23.37 ± 0.96 g were hydrodinamically gene transferred by administering into the tail vein a bolus with ICL and MS. After 36 hours, body weight, plasma glucose, respiratory quotient and energy expenditure were measured. The respiratory quotient was increased by gene transfer, which suggests that a higher carbohydrate/lipid ratio is oxidized in such animals. This application could help, if adequate protocols are designed, to induce fat utilization for glucose synthesis, which might be eventually useful to reduce body fat depots in situations of obesity and diabetes.

## Background

Thousands of different life-related biochemical processes, such as cell respiration and many other metabolic reactions, can lead to the production and utilization of energy in forms of ATP synthesis and heat release [1-3]. Indeed, all biological processes, including the chemical pathways concerning bioenergetics, follow thermodynamic laws [4].

The central set of reactions involved in cellular fuel homeostasis are collectively known as the citric acid or tricarboxilic acid cycle (TCA cycle), which oxidizes the products of glycolisis and lipid-derived substrates, such as acetyl-CoA, to produce energy in the mitochondria [1]. Indeed, mitochondrial damage and the subsequent dysfunction in this membrane-enclosed organelle are often manifested as neurological disorders, but also as diabetes or obesity [5].

In this context, the glyoxylate cycle is a metabolic pathway well characterized in plants, fungi and several microorganisms [6]. Interestingly, the glyoxylate cycle allows these organisms to use fats for the synthesis of carbohydrates via the acetate generated during fatty acid β-oxidation, which is achieved by two unique enzymes: isocitrate lyase (ICL; EC 4.1.3.1) and malate synthase (MS; EC 2.3.3.9). These enzymes sequentially catalyse chemical conversions, which are involved in the glyoxylate cycle and appear to be absent or unfunctional in most circumstances in vertebrates, including rodents [7-9], guinea pigs [10] and humans [11].

Current gene transfer methodologies are able to incorporate selected nucleotide sequences into the nucleus of target cells [12,13] by means of naked DNA or encoded by molecular constructs named vectors, which can be viral (mainly adeno or retrovirus) or non-viral (lipoplexes, dendromers and others) in order to activate some metabolic linked processes with potential application in gene therapy [14-16].

Considering these findings and observations, our aim was to ascertain the viability of a hydrodynamic gene transfer [17,18] to achieve the heterologous expression of ICL and MS in mouse hepatocytes, in order to produce a bypass in the tricarboxilic acid cycle, enabling the carbons derived from fatty acid oxidation to be preserved and converted into glucose (gluconeogenesis). This approach generating a flow from cell lipid reserves to carbohydrate utilization, can be appropriately assessed after hydrodynamic transfer of two glyoxylate cycle enzyme genes by measuring the respiratory quotient [19,20], as an indicator of the macronutrient mixture oxidized and indirectly the fat-to-glucose conversion.

## Methods

Plasmids. DNA codifying both bacterial enzymes of the glyoxylate cycle (ICL and MS) was obtained from ATCC and both concerned the *Vibrio cholearae *sequence gb/AE003852.1/from 01 biovar eltor str N16961 chromosome 1 (clones GVCJA51 for ICL and GVCDP57 for MS). Both clones were subcloned by PCR and inserted in the BssHI restriction enzyme region of the commercial plasmid pCMV/myc/mito (Invitrogen, USA). Later, they were transformed in *Escherichia coli *for laboratory scale production using a commercial kit from Qiagen (USA). The plasmid integrity was checked by enzymatic digestion and subsequent agarose gel.

Animals. Twenty male C57BL6J mice (Harlan, Italy) of about 23 g were kept at 21–23°C, 50 ± 10% humidity on a 12:12 light-dark cycle (8:00–20:00 h.). After a period of acclimatation of seven days in individual cages, the animals were weighed and assigned to two dietary groups for 36 additional hours: one group (C, n = 10) received *ad libitum *commercial food (Harlan Iberica 2014S) containing 349 Kcal/100 g (73% carbohydrates, 10% lipids and 17% protein), and the other group (F, n = 10) was fasted for the same period. Free water was available at any time in all cages.

Half of the animals assigned to every dietary group randomly received a hydrodynamic load with the control plasmid pCMV/myc/mito (C and F, n = 5) or both plasmids containing the two glyoxylate enzymes (CGx and FGx, n = 5). Thus, the mice were administered, in approximately 6 seconds, with a bolus injection in the tail vein with 10% of their weights (g) in volume (ml) of a complex containing MIRUS polimer solution (Madison, USA) and approximately 20 mg of DNA according to the manufacturer's instructions (MIRUS: MIR-100 trans IT^® ^in vivo gene delivery system) at a constant rate. An equimolar ratio (2:1+1 mols) was used for administering a bolus containing pCMV/myc/mito or pCMV/myc/mito-ICL and pCMV/myc/mito-MS into the animals. The success of the process was assessed by repeating the protocol by measuring β-galactosidase staining of Lac Z transgene and luciferase activity after CMV-Luc and Lac Z gene hydrodynamic administration in mice under similar conditions [21].

After 36 hours, body weights were carefully measured and final glucose was assessed with a digital glycometer (Medisense Optium, UK) from the jugular vein. The respiratory quotient was measured by using an indirect calorimeter for two hours before the mice were sacrificed. All animal manipulations were made in accordance with European Community Guidelines and University of Navarra Ethical Committee for the use of laboratory animals.

Indirect calorimetry. Oxygen (O_2_) consumption, carbon dioxide (CO_2_) production and respiratory quotient (RQ) were measured using an Oxylet 00 O_2_/CO_2 _indirect calorimeter (Panlab SL, Spain) [22] following manufacturer's instructions and using an appropriate software (Chart, ADIntruments, Australia). The O_2 _and CO_2 _analyzers were calibrated with highly purified gas standards (Praxair, Spain) and each animal was placed into one of four acrylic chambers (140 mm diameter × 150 mm height each). Room air was drawn through each chamber at a rate of 300 mL/min. The O_2 _and CO_2 _levels were then measured on 3-min sampling periods (8 sampling periods per 2 hours of data collection with three chambers) to generate difference scores between data derived from each mouse chamber versus the data collected from room air [22]. RQ was calculated as the ratio of the volume of CO_2 _produced by the volume of O_2 _consumed, while energy expenditure was calculated according to the formula [19,23]: EE (kcal/day/body weight^3/4^) = O_2 _volume × 1.44 × [3.815 + (1.232 × RQ)].

## Results and discussion

**Figure 1:**
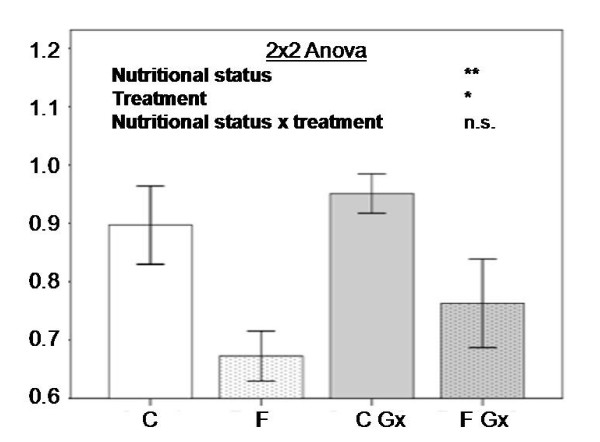
Means ± SD of respiratory quotient and differences analized by 2 × 2 ANOVA (nutritional status × treatment). (C; control diet, F; fasting group, C Gx; control diet group with full plasmid, F Gx; fasting group with full plasmid, n.s.; non statistical significance, *; p < 0.05, **; p < 0.01).

As expected, body weight and plasma glucose measurements were affected by the 36-hour fasting period, being statistically lower in the food-deprived mice (table 1). The administration of both plasmids (ICL+MS) or the Control one produced similar changes in both variables (body weight and glycemia) within each nutritional group (control fed or fasted). Energy expenditure values were apparently unaffected by the treatments when they were normalized by body weight. Interestingly, those fasted mice receiving both ICL+MS plasmids showed an increased (p < 0.05) respiratory quotient (figure 1) as compared to the fasted control group (0.76 ± 0.08 vs. 0.67 ± 0.05). This trend was also observed (p < 0.05) in the control fed group compared to the ICL+MS plasmids-administered mice (0.90 ± 0.08 vs. 0.95 ± 0.04) (figure 1).

**Table 1:** 

					2 × 2 Anova		
	C (n = 5)	F (n = 5)	C Gx (n = 5)	F Gx (n = 5)	Nutritional Status	Treatment	Nutritional status × treatment
Weight change (g)	1.47 ± 2.59	-4.53 ± 2.28	0.07 ± 1.14	-3.33 ± 1.73	**	n.s.	n.s.
Final weight (g)	24.8 ± 2.59	18.8 ± 2.28	23.4 ± 1.14	20.0 ± 1.73	**	n.s.	n.s.
Glucose (mg/dL)	112.8 ± 16.5	75.6 ± 30.2	122.4 ± 28.6	69.2 ± 33.9	**	n.s.	n.s.

Means ± SD of body weight change (g), final weight (g) and plasma glucose (mg/dL) analized by 2 × 2 ANOVA (nutritional status × treatment). (C; control diet, F; fasting group, C Gx; control diet group with full plasmid, F Gx; fasting group with full plasmid, n.s.; non statistical significance, **; p < 0.01).

The results of this experimental trial confirm that hydrodynamic based gene transfer technology is able to increase the RQ, which suggests that more glucose is oxidized in relation to lipid utilization [19,20]. This finding was especially relevant in the fasting situation, which is interesting because in such conditions lipid reserves are more required for energy expenditure maintenance. The trends in the fed group were also in the same direction, confirming the validity of this gene therapy tool.

The glyoxylate cycle is a critical component of the gluconeogenic machinery responsible for the conversion of acetate into glucose [6]. Indeed, acetate can only serve as a net source of glucose in organisms with the necessary enzymes to catalyze the reactions of the glyoxylate cycle [24]. In this context, ICL and MS are recognized as the pathway specific enzyme activities, while no remaining enzyme activities were in common with activities of the TCA cycle [25].

The glyoxylate cycle is well characterized in microorganisms, higher plants [26] and nematods, but the occurrence and functionality in vertebrates, such as rodents, chickens or humans [7-11], is still a matter of debate. This has been attributed to the lack of this metabolic pathway, reduced expression of ICL/MS in normal conditions, or improper induction or measurement of the activity of these enzymes [27].

In this context, our hypothesis was that the presence of a functional glyoxylate cycle might enable the organism to obtain more energy supply from sources different from glucose and facilitate fat mobilization. Strategies based on this approach might accelerate fat utilization in fasting situations or during exercise, increasing thus fuel demands, which might be very useful in obesity and diabetes to reduce adipose tissue depots.

Despite the apparent success of this gene transfer, some aspects and limitations should be taken into account, such as the administered dose, which may affect liver function, as described by others [17,18], the limited number of animals in this translational research or the short period of time analyzed given that fasting can not be maintained at long term [27], among others.

Summing up, this pioneer protocol was able to induce changes in the mechanism of secondary glucose/glycogen production, by the conversion of fat to carbohydrates allowing the net utilization of sugars from acetyl-CoA. This approach, if successfully developed in other species including the human being, could provide a valid gene therapy instrument for obesity and related comorbilities, in which accelerated fat oxidation is required.

## Abbreviations

ICL: Isocytrate lyase; MS: Malate synthase; TCA cycle: Tricarboxilic acid cycle; RQ: Respiratory quotient; ATCC: American type culture collection.

## Competing interests

The authors declare that they have no competing interests.

## Authors' contributions

JC, FIM and JAM conceived and designed the experiments; JC, FIM and PC performed the experiments; PC performed the statistical analyse; JC, FIM, PC, and JAM wrote and edited the manuscript; FM and JAM assumed the economic management.
